# Functional Dichotomy for a Hyphal Repressor in Candida albicans

**DOI:** 10.1128/mbio.00134-23

**Published:** 2023-03-08

**Authors:** Yinhe Mao, Norma V. Solis, Scott G. Filler, Aaron P. Mitchell

**Affiliations:** a Department of Microbiology, University of Georgia, Athens, Georgia, USA; b Lundquist Institute for Biomedical Innovation at Harbor-UCLA Medical Center, Torrance, California, USA; c David Geffen School of Medicine at UCLA, Los Angeles, California, USA; Universidade de Sao Paulo

**Keywords:** *Candida albicans*, gene regulation, genetics, hyphal development, natural variation

## Abstract

Nrg1 is a repressor of hypha formation and hypha-associated gene expression in the fungal pathogen Candida albicans. It has been well studied in the genetic background of the type strain SC5314. Here, we tested Nrg1 function in four other diverse clinical isolates through an analysis of *nrg1*Δ/Δ mutants, with SC5314 included as a control. In three strains, *nrg1*Δ/Δ mutants unexpectedly produced aberrant hyphae under inducing conditions, as assayed by microscopic observation and endothelial cell damage. The *nrg1*Δ/Δ mutant of strain P57055 had the most severe defect. We examined gene expression features under hypha-inducing conditions by RNA-sequencing (RNA-Seq) for the SC5314 and P57055 backgrounds. The SC5314 *nrg1*Δ/Δ mutant expressed six hypha-associated genes at reduced levels compared with wild-type SC5314. The P57055 *nrg1*Δ/Δ mutant expressed 17 hypha-associated genes at reduced levels compared with wild-type P57055, including *IRF1*, *RAS2*, and *ECE1*. These findings indicate that Nrg1 has a positive role in hypha-associated gene expression and that this role is magnified in strain P57055. Remarkably, the same hypha-associated genes affected by the *nrg1*Δ/Δ mutation in strain P57055 were also naturally expressed at lower levels in wild-type P57055 than those in wild-type SC5314. Our results suggest that strain P57055 is defective in a pathway that acts in parallel with Nrg1 to upregulate the expression of several hypha-associated genes.

## OBSERVATION

Genotype-phenotype connections invariably depend upon genetic background (1–4). The mutant phenotype helps define the biological function of a gene, and thus, the genetic background used in a study can affect interpretation profoundly. Conversely, background effects can be exploited to reveal informative genetic impacts in one strain that are cryptic in another (5).

Our focus is the fungal pathogen Candida albicans, a human commensal that causes both mucosal and deep tissue infections in susceptible individuals (6). Almost all molecular studies of C. albicans have employed the SC5314 strain background. Benefits of a type strain abound, and yet, the analysis of other clinical isolates has repeatedly offered novel insight into drug resistance, pathogenicity, and basic biology (7–10).

Among the most well-studied C. albicans virulence traits is the ability to produce hyphae, which are long cylindrical cells that grow by tip extension and remain attached after division (11). Hyphal impact comes from the novel morphology and the expression of hypha-associated genes, whose products include cell surface proteins, secreted proteases, and the secreted Candidalysin toxin (12, 13).

Hypha production and hypha-associated gene expression are regulated by a large network of transcription factors (TFs) (14–16). These TFs include both positive and negative regulators of hypha-associated genes. Recent studies of positive regulators in diverse C. albicans clinical isolates indicate that phenotypic output can vary dramatically among strains (17, 18). For the master hyphal regulator Efg1, strain-limited expression levels of interacting TFs shape the effects of Efg1 on target gene expression (17).

Here, we focus on the negative hyphal regulator Nrg1. Nrg1 represses hypha-associated genes under yeast growth conditions (19, 20). Therefore, *nrg1*Δ/Δ mutants produce hyphae under noninducing growth conditions. Under hypha-inducing conditions, *nrg1*Δ/Δ mutants in the SC5314 strain background form apparently normal hyphae (19, 20).

**FIG 1:**
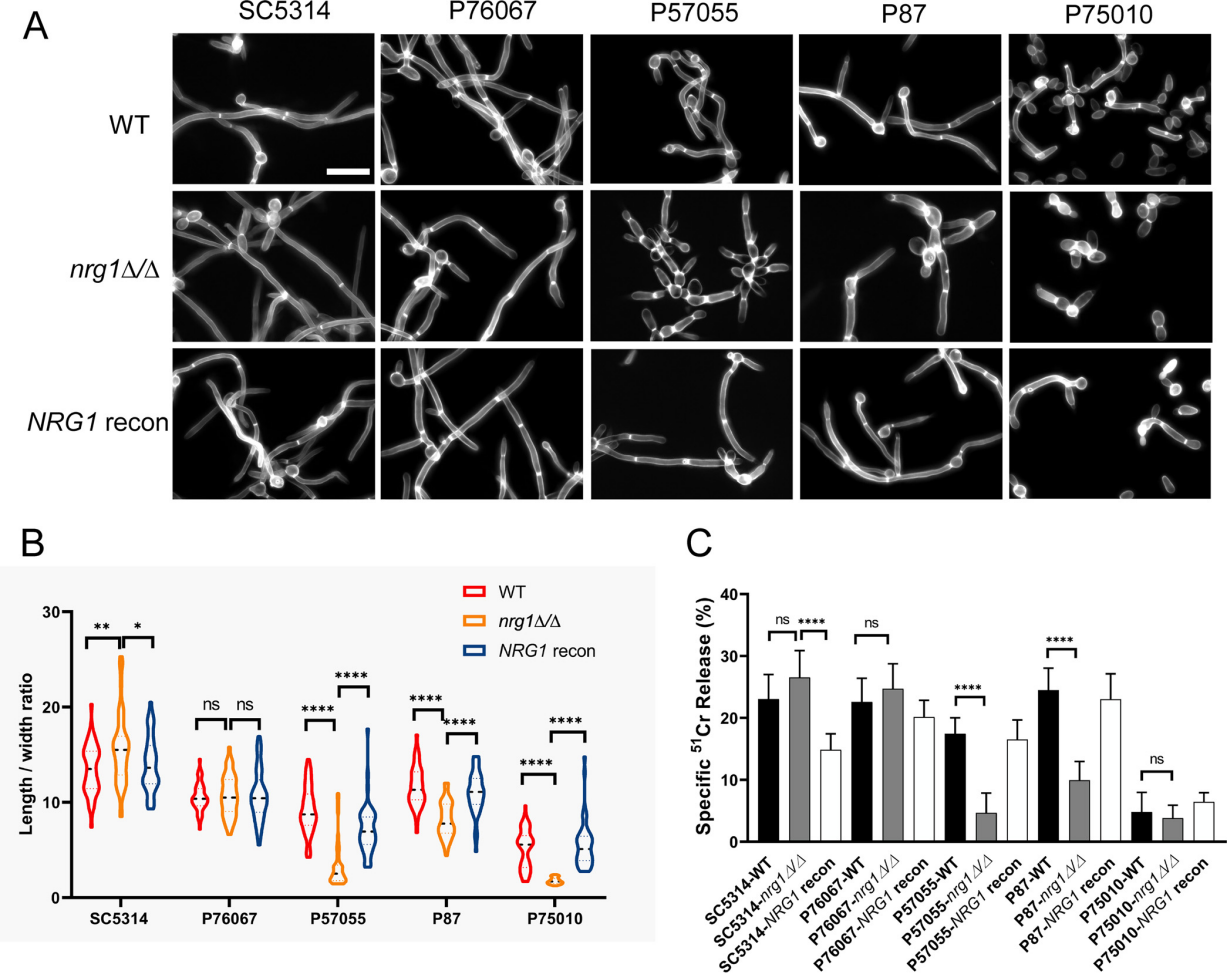
*NRG1* mutant phenotypes. Wild-type, *nrg1*Δ/Δ mutant, and *NRG1* reconstituted strains were constructed in five C. albicans clinical isolate backgrounds, as follows: SC5314 (clade 1), P76067 (clade 2), P57055 (clade 3), P87 (clade 4), and P75010 (clade 11) following standard methods (Text S1). Genotypes, plasmids, and primer sequences are listed in Table S1. (A) Filamentation assays. Strains were grown in YPD medium overnight at 30°C with shaking and transferred to RPMI with 10% serum at 37°C for 4 h. Fixed cells were stained with calcofluor white for confocal microscopy. White scale bar, 20 μm. (B) Length/width ratios. Cell unit features from the experiment in A were quantified with ImageJ, using a minimum of 100 cells and 3 fields. Values are means with SD. Data were analyzed with the Tukey-Kramer test; ns, *P* > 0.05; *, *P* < 0.05; **, *P* < 0.01; ****, *P* < 0.0001. (C) Endothelial cell damage capability. Human endothelial cells were incubated with the indicated strains of C. albicans for 3 h, after which the extent of host cell damage was determined using a ^51^Cr release assay, following standard methods (21). Results are the mean of three independent experiments, with each performed in triplicate. Error bars denote standard deviation. Data were analyzed with the Tukey-Kramer test; ns, *P* > 0.05; ****, *P* < 0.0001.

We constructed *nrg1*Δ/Δ mutants and reconstituted derivatives (see Table S1 and Text S1 in the supplemental material) in five diverse C. albicans clinical isolates (8, 18). As expected, the mutants all produced polarized cells or hyphae under noninducing conditions (in yeast extract-peptone-dextrose [YPD] at 30°C) (see Fig. S1 in the supplemental material). Surprisingly, though, hypha formation was aberrant under strongly inducing conditions (in RPMI + fetal bovine serum [FBS] at 37°C) (Fig. 1A and B) in the mutants of strains P57055, P87, and P75010. The defect was reflected quantitatively in a reduced cell length/width ratio (Fig. 1B). The defect was caused by the *nrg1*Δ/Δ mutation rather than a secondary mutation because it was reversed in reconstituted strains (Fig. 1A and B). These results indicate that Nrg1 has a positive role in hypha formation in some C. albicans isolates.

10.1128/mbio.00134-23.1 FIG S1 Cell morphology during growth under noninducing conditions (in YPD at 30°C). White scale bar, 20 μm. Download FIG S1, PDF file, 0.9 MB. Copyright © 2023 Mao et al. 2023 Mao et al. https://creativecommons.org/licenses/by/4.0/ This content is distributed under the terms of the Creative Commons Attribution 4.0 International license.

10.1128/mbio.00134-23.2 TABLE S1 Strains, plasmids, and primers used in this study. Download Table S1, XLSX file, 0.01 MB. Copyright © 2023 Mao et al. 2023 Mao et al. https://creativecommons.org/licenses/by/4.0/ This content is distributed under the terms of the Creative Commons Attribution 4.0 International license.

10.1128/mbio.00134-23.5 TEXT S1 Detailed methods. Download Text S1, DOCX file, 0.04 MB. Copyright © 2023 Mao et al. 2023 Mao et al. https://creativecommons.org/licenses/by/4.0/ This content is distributed under the terms of the Creative Commons Attribution 4.0 International license.

Host cell damage capability is a functional output of hypha formation (21). The *nrg1*Δ/Δ mutation caused no defect in endothelial cell damage in strains SC5314 or P76067 (Fig. 1C). However, the mutation caused a significant damage defect in strains P57055 and P87. The mutation caused no significant defect in strain P75010, although the weak damage capability of that wild type (WT) may have precluded the detection of a defect. The results indicate that Nrg1 is required for both function and morphogenesis of hyphae in strains P57055 and P87.

We sought to understand how Nrg1 may promote hypha formation in some strains through RNA-sequencing (RNA-Seq) analysis (see Table S2 in the supplemental material) (NCBI BioProject accession number PRJNA925154). We compared *nrg1*Δ/Δ versus wild-type strains in the SC5314 and P57055 backgrounds under growth conditions (RPMI + FBS at 37°C for 4 h) that yielded apparently normal hyphae in SC5314 but not in P57055. The SC5314 *nrg1*Δ/Δ mutant profile included 29 downregulated RNAs and 252 upregulated RNAs, in keeping with the major activity of Nrg1 as a repressor (22). Downregulated genes were weakly enriched for adhesion functions; upregulated genes were greatly enriched for carbohydrate transport functions (see Table S3 in the supplemental material). The P57055 *nrg1*Δ/Δ mutant profile included 77 downregulated RNAs and 187 upregulated RNAs. Downregulated genes were enriched for biofilm functions; upregulated genes were again greatly enriched for carbohydrate transport functions (Table S3). Therefore, the broad kinds of functions affected by an *nrg1*Δ/Δ mutation are similar in the two backgrounds.

10.1128/mbio.00134-23.3 TABLE S2 Comparisons among RNA-Seq data sets for P57055, P57055 *nrg1*Δ/Δ, SC5314, and SC5314 *nrg1*Δ/Δ. Raw data are available at the NCBI (BioProject ID PRJNA925154). Download Table S2, XLSX file, 1.3 MB. Copyright © 2023 Mao et al. 2023 Mao et al. https://creativecommons.org/licenses/by/4.0/ This content is distributed under the terms of the Creative Commons Attribution 4.0 International license.

10.1128/mbio.00134-23.4 TABLE S3 Gene ontology process features of upregulated and downregulated genes in *nrg1*Δ/Δ mutants. Download Table S3, XLSX file, 0.02 MB. Copyright © 2023 Mao et al. 2023 Mao et al. https://creativecommons.org/licenses/by/4.0/ This content is distributed under the terms of the Creative Commons Attribution 4.0 International license.

**FIG 2:**
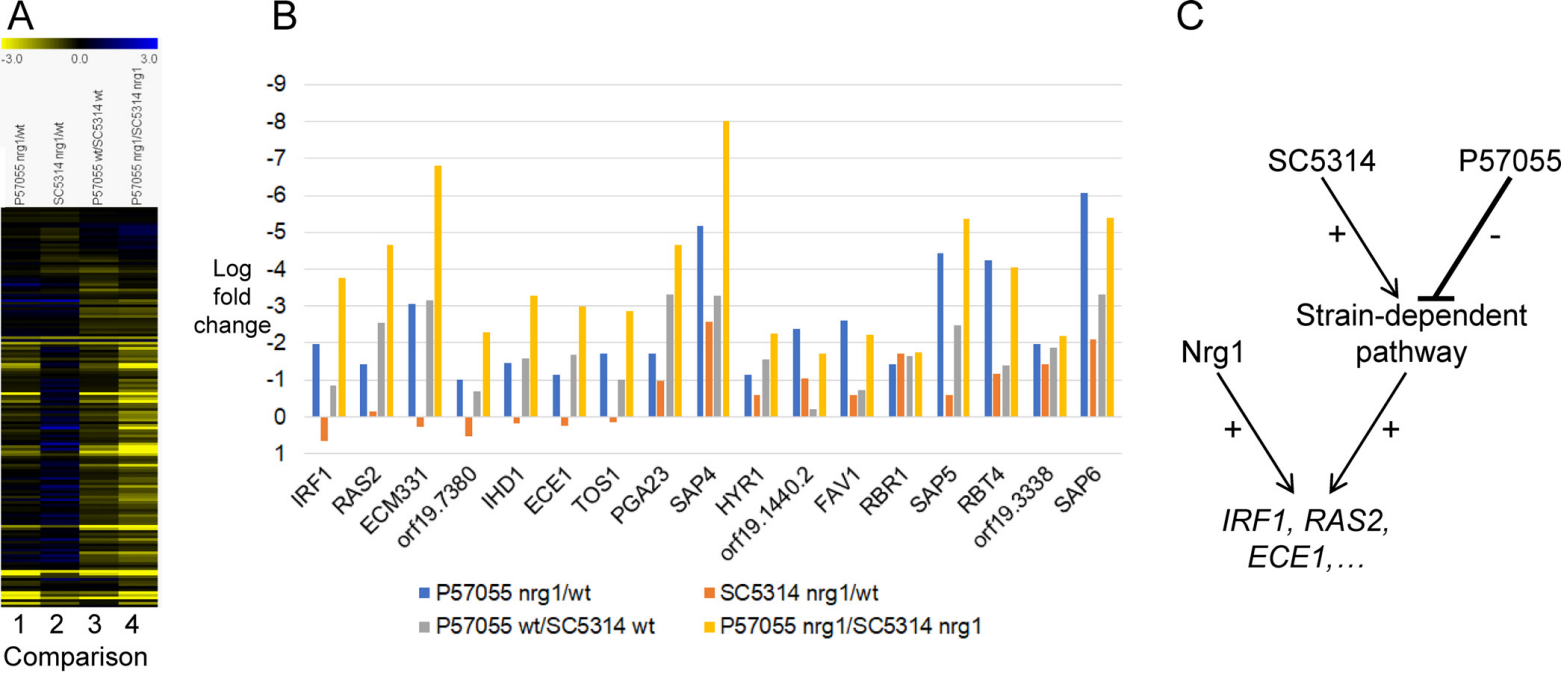
Gene expression analysis of *nrg1*Δ/Δ mutants. Cells were grown in RPMI + 10% FBS for 4 h at 37°C, and RNA was extracted as previously described (30). RNA-Seq, read mapping, and differential expression analysis using the DESeq2 R package (1.14.1) were performed by Novogene (Sacramento, CA), using three biological replicates per group (Text S1). (A) Heat map representation of expression differences in 152 hypha-associated genes (23). Comparisons include P57055 *nrg1*Δ/Δ versus P57055 WT (comparison 1), SC5314 *nrg1*Δ/Δ versus SC5314 WT (comparison 2), P57055 WT versus SC5314 WT (comparison 3), and P57055 *nrg1*Δ/Δ versus SC5314 *nrg1*Δ/Δ (comparison 4). The color scale covers the range −3log_2_ (yellow, downregulated) to 3log_2_ (blue, upregulated). (B) Expression changes for 17 hypha-associated genes that were differentially expressed in the P57055 *nrg1*Δ/Δ mutant compared with P57055 wild type (log fold change, >2; adjusted *P* < 0.05) (Table S2). The *y* axis has been inverted for ease of visualization. RNA-Seq data are available at the NCBI (BioProject PRJNA925154) and in Table S2. (C) Model for positive control of target genes by Nrg1 at 37°C. In strain SC5314, key target genes (e.g., *IRF1* and *RAS2*) are under positive control by a strain-dependent pathway—one that is active in some strains but not others. This pathway functions independently of Nrg1, and hence, an *nrg1*Δ/Δ mutation has little effect on target gene expression. In strain P57055, the strain-dependent pathway is inactive. For that reason, target gene expression is slightly reduced in wild-type P57055 relative to wild-type SC5314. In addition, target gene expression is more dependent on Nrg1 in P57055, and the *nrg1*Δ/Δ mutation causes a pronounced defect in hypha formation.

We focused specifically on hypha-associated genes to determine whether the *nrg1*Δ/Δ mutation affected these genes differently in the two strain backgrounds. We used a set of 152 genes derived from comparisons of strongly and weakly filamentous C. albicans strains (23). The P57055 *nrg1*Δ/Δ mutant had downregulated RNA levels for 17 of the genes (Fig. 2A, comparison 1; Table S2); the SC5314 *nrg1*Δ/Δ mutant had downregulated RNA levels for only 6 genes (Fig. 2A, comparison 2; Table S2). Many hypha-associated genes were also downregulated in wild-type P57055 compared with those of wild-type SC5314 (Fig. 2A, comparison 3; Table S2). Remarkably, though, the same genes that were affected by the *nrg1*Δ/Δ mutation in P57055 were also affected by the P57055 genetic background (Fig. 2A, comparisons 1 and 3). For example, *IRF1* was downregulated 4-fold by the *nrg1*Δ/Δ mutation in P57055 and expressed at 2-fold lower levels in wild-type P57055 compared with those in SC5314 (Fig. 2B; Table S2). Also, *RAS2* was downregulated 3-fold by the *nrg1*Δ/Δ mutation in P57055 and expressed at 5-fold lower levels in wild-type P57055 compared with those of SC5314 (Fig. 2B; Table S2). Also notable was that *ECE1* was downregulated 2-fold by the *nrg1*Δ/Δ mutation in P57055 and expressed at 3-fold lower levels in wild-type P57055 compared with those in SC5314 (Fig. 2B; Table S2). The net result was that several hypha-associated genes were expressed at 8- to 100-fold lower levels in the P57055 *nrg1*Δ/Δ mutant than in the SC5314 *nrg1*Δ/Δ mutant (Fig. 2A, comparison 4; Fig. 2B; Table S2). Therefore, P57055 naturally expresses several Nrg1-activated genes at lower levels than SC5314, and the P57055 background effects are augmented by an *nrg1*Δ/Δ mutation.

The expression defects of the P57055 *nrg1*Δ/Δ mutant help explain its hyphal defect. *IRF1* and *RAS2* both have positive roles in hypha formation (24, 25), so their reduced expression may contribute to the hyphal morphogenesis defect. *ECE1* encodes the Candidalysin precursor (26), so its reduced expression may contribute to the host cell damage defect. Background effects often reflect interactions among multiple genes (1, 2, 27). For that reason, it is likely that additional differences in alleles or gene expression levels also contribute to the divergent behavior of P57055 and SC5314 *nrg1*Δ/Δ mutants.

These gene expression comparisons suggest that P57055 may be defective in a pathway that acts in parallel to Nrg1 to affect the expression of some of the same genes (Fig. 2C). In SC5314, where the pathway functions efficiently, an *nrg1*Δ/Δ mutation causes little if any reduction in hypha-associated gene expression. In P57055, where the pathway functions poorly, an *nrg1*Δ/Δ mutation causes a prominent reduction in hypha-associated gene expression.

Which gene products act in the strain-dependent pathway? Mutant alleles found in natural populations are typically different from and less severe than null alleles (2), and strain variation generally reflects interactions among multiple alleles (28). Therefore, causal mutations that inactivate the pathway in P57055 may be difficult to identify from nucleotide sequences. Our RNA-Seq data provide some candidate genes that may function in the pathway (Table S2). For example, *UME6*, which specifies a positive regulator of hypha-associated genes (29), is expressed at lower levels in P57055 than that in SC5314. Also, seven *TLO* genes are expressed at lower levels in P57055 than those in SC5314. The telomeric *TLO* gene family specifies Med2 mediator subunits and several impact hypha formation (30, 31). The *UME6* and *TLO* gene products may act in the strain-dependent pathway to mask the positive role of Nrg1 in hypha formation in strain SC5314 and reveal it in strain P57055.

### Data availability.

RNA-Seq data are available at the NCBI (BioProject PRJNA925154) and in Table S2.
