# Supplementary figures and images for: Functional Dichotomy for a Hyphal Repressor in Candida albicans
Source: mBio. 2023 Mar 8;14(2):e00134-23. doi: 10.1128/mbio.00134-23 (PMC10127614; doi:10.1128/mbio.00134-23)

**Figure S1.** Cell morphology during growth under non-inducing conditions (YPD, 30°C). White scale bar, 20  $\mu$ m.

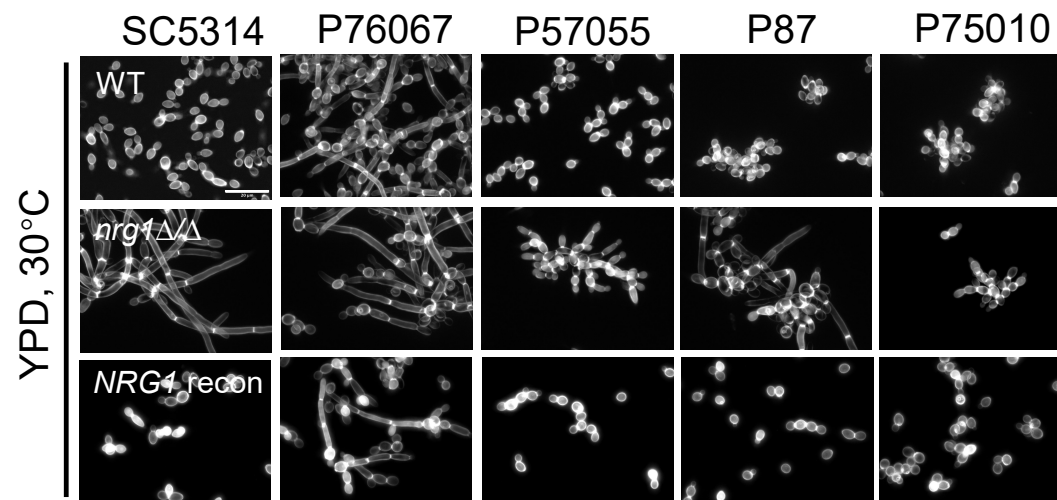

Supplement: FIG S1 [file mbio.00134-23-s0001.pdf]
