# Supplementary material for: Functional Dichotomy for a Hyphal Repressor in Candida albicans
Source: mBio. 2023 Mar 8;14(2):e00134-23. doi: 10.1128/mbio.00134-23 (PMC10127614; doi:10.1128/mbio.00134-23)
Supplement: TEXT S1 [file mbio.00134-23-s0005.docx]

**METHODS**

**Strains and media.** All strains are listed in Table S1. *C. albicans* strains SC5314, P76067, P57055, P87 and P75010 and their derived *his1∆/∆* mutants were used as transformation recipients (1). Fungal strains were grown at 30°C in YPD (2% Bacto peptone, 2% dextrose, 1% yeast extract) with shaking. For phenotypic assays, strains were grown either in liquid RPMI 1640 medium (Sigma-Aldrich, St. Louis, MO) adjusted to pH 7.4 and supplemented with 10% fetal bovine serum (Atlanta Biologicals, Inc., Flowery Branch, GA) or in YPD medium. *C. albicans* transformants were selected on CSM-HIS (0.67% Yeast Nitrogen Base without amino acids, 0.074% CSM-HIS and 2% dextrose) for His+ isolates or YPD plus NAT (2% Bacto peptone, 2% dextrose, 1% yeast extract and 400 mg/mL [Werner BioAgents]) for nourseothricin-resistant (NatR) isolates. All strains were stored as glycerol stocks at -80°C.

**Plasmids and DNA.** Primers and plasmids are listed in Table S1.

**Deletion strain construction.** Construction of the *nrg1∆/∆* mutants in clinical isolates was employed as described previously (1, 2). Briefly, there were 3 steps. (a) Construction of sgRNA expression cassette. The *SNR52* promoter was amplified with primers SNR52/F and SNR52/R_NRG1 from plasmid pV1093 (3); the sgRNA scaffold was amplified with primers sgRNA/F_NRG1 and sgRNA/R from plasmid pV1093. Then fusion PCR with nested primers SNR52/N and sgRNA/N was used to amplify the final sgRNA expression cassette. (b) Construction of *nrg1∆::r1HIS1r1* cassette. One of the two halves of the *nrg1∆::r1HIS1r1* cassette was amplified from pMH01 using primers His1 CRIME/F and NRG1 CRIME adapR; the other half of the *nrg1∆::*r1HIS1r1 cassette was amplified from pMH02 using primers His1 CRIME/R and NRG1 CRIME adapF. (c) The Cas9 cassette was amplified from the plasmid pV1093 (3) by primers CaCas9/for and CaCas9/rev. Cas9 cassette was transformed with NRG1 sgRNA cassette and *nrg1∆::r1HIS1r1* cassette to five his1- isolates via the transient CRISPR-Cas9 system. Transformants were verified by primers NRG1 Check/F and CdHIS1 Check int/R; primers NRG1 Check/F and NRG1 Check int/R.

**Complemented strain construction** We constructed the reconstituted strains by reintroducing a copy of the SC5314 allele of *NRG1* at the *NRG1* deletion locus of all *nrg1∆/∆* mutants using our concatemer assembly method (2). First, an *NRG1* cassette was amplified from SC5314 genome containing an SC5314 *NRG1* allele using primers NRG1 check/F and NRG1 3'R->pNAT5’/R, containing concatenating homology to a NAT1 marker. Then the *NAT1* marker was amplified from pNAT using pNAT/F and pNAT 3'R ->NRG1 down/R. The *NRG1*-containing cassette, corresponding *NAT1* marker and r1 sgRNA DNA cassette were transformed into the *nrg1*Δ/Δ mutant strains in all clinical isolate backgrounds. The transient CRISPR-Cas9 system was employed as previously described in detail (4). Generally, the Cas9 cassette was amplified from the plasmid pV1093, and each of sgRNA cassette was generated by using split-joint PCR with “sgRNA/F r1” and “SNR52/R r1”.

**Filamentation assay.** To assay hypha formation, strains were inoculated from YPD overnight cultures to an OD_600_ of 0.4 into 5 mL of RPMI with 10% serum in glass test tubes. Cells were grown for 4 h at 37°C with shaking, then collected by centrifugation and fixed with 4% formaldehyde for 15 min. Fixed cells were washed in phosphate-buffered saline (PBS), stained with 200 ng/mL calcofluor white, and imaged using a slit-scan confocal optical unit on a Zeiss Axiovert 200 microscope with a Zeiss C- Apochromat 63x, 1.2 numerical aperture oil immersion objective. Lengths / widths of hyphal units, i.e., the distance between septa on hyphae, were quantified using ImageJ. At least 100 interseptal distance measurements were taken from 3 separate views.

**RNA extraction.** RNA extractions were done according to the previously described method (5). Briefly, Cells grown in 5 mL YPD for overnight at 30°C were washed and then cultured in 25 mL of RPMI plus 10% FBS for 4 hours at 37°C. RNA extraction was performed using a Qiagen RNeasy mini kit (Cat#74104) with some modifications.

**RNA sequencing.** A total amount of 1 μg RNA per sample was used as input material for the RNA-seq sample preparations. Sequencing libraries were generated using NEBNext® UltraTMRNA Library Prep Kit for Illumina® (NEB, USA). 150 nt of sequence was determined from both ends of each cDNA fragment using the Illumina platform. Sequencing reads were aligned to the *C. albicans* reference (Assembly A21) using HISAT2. Differential expression analysis between two groups (three biological replicates per group) was performed using the DESeq2 R package (1.14.1).

**Endothelial cell damage assay.** Endothelial cell damage by *C. albicans* cells was assessed as previously described (6). Briefly, human endothelial cells were grown in a 96-well tissue culture plate containing detachable wells and loaded with ^51^Cr overnight. Cells were washed, and inoculated with each strain cells at a concentration of 4×10^4^ organisms per well. Cells were incubated for 3 h and the ^51^Cr release was quantified using a gamma counter.

**Data availability.** RNA-Seq data are available through NCBI BioProject accession number PRJNA925154 in the SRA database.

**REFERENCES**

1. Huang MY, Woolford CA, May G, McManus CJ, Mitchell AP. 2019. Circuit diversification in a biofilm regulatory network. PLoS Pathog 15:e1007787.

2. Huang MY, Woolford CA, Mitchell AP. 2018. Rapid Gene Concatenation for Genetic Rescue of Multigene Mutants in Candida albicans. mSphere 3.

3. Vyas VK, Barrasa MI, Fink GR. 2015. A Candida albicans CRISPR system permits genetic engineering of essential genes and gene families. Sci Adv 1:e1500248.

4. Min K, Ichikawa Y, Woolford CA, Mitchell AP. 2016. Candida albicans Gene Deletion with a Transient CRISPR-Cas9 System. mSphere 1.

5. Cravener MV, Mitchell AP. 2020. Candida albicans Culture, Cell Harvesting, and Total RNA Extraction. Bio Protoc 10:e3803.

6. Sanchez AA, Johnston DA, Myers C, Edwards JE, Jr., Mitchell AP, Filler SG. 2004. Relationship between Candida albicans virulence during experimental hematogenously disseminated infection and endothelial cell damage in vitro. Infect Immun 72:598-601.
